# Diverse Genomic Traits Differentiate Sinking-Particle-Associated versus Free-Living Microbes throughout the Oligotrophic Open Ocean Water Column

**DOI:** 10.1128/mbio.01569-22

**Published:** 2022-07-12

**Authors:** Andy O. Leu, John M. Eppley, Andrew Burger, Edward F. DeLong

**Affiliations:** a Daniel K. Inouye Center for Microbial Oceanography: Research and Education (C-MORE), University of Hawaii, Honolulu, Hawaii, USA; Rutgers, The State University of New Jersey

**Keywords:** carbon pump, deep sea, free-living, marine microbes, particle-attached

## Abstract

Bacteria and archaea are central to the production, consumption, and remineralization of dissolved and particulate organic matter and contribute critically to carbon delivery, nutrient availability, and energy transformations in the deep ocean. To explore environmentally relevant genomic traits of sinking-particle-associated versus free-living microbes, we compared habitat-specific metagenome-assembled genomes recovered throughout the water column in the North Pacific Subtropical Gyre. The genomic traits of sinking-particle-associated versus free-living prokaryotes were compositionally, functionally, and phylogenetically distinct. Substrate-specific transporters and extracellular peptidases and carbohydrate-active enzymes were more enriched and diverse in particle-associated microbes at all depths than in free-living counterparts. These data indicate specific roles for particle-attached microbes in particle substrate hydrolysis, uptake, and remineralization. Shallow-water particle-associated microbes had elevated genomic GC content and proteome nitrogen content and reduced proteome carbon content in comparison to abyssal particle-associated microbes. An inverse trend was observed for their sympatric free-living counterparts. These different properties of attached microbes are postulated to arise in part due to elevated organic and inorganic nitrogen availability inside sinking particles. Particle-attached microbes also were enriched in genes for environmental sensing via two-component regulatory systems, and cell-cell interactions via extracellular secretion systems, reflecting their surface-adapted lifestyles. Finally, particle-attached bacteria had greater predicted maximal growth efficiencies than free-living bacterioplankton at all depths. All of these particle-associated specific genomic and proteomic features appear to be driven by microhabitat-specific elevated nutrient and energy availability as well as surface-associated competitive and synergistic ecological interactions. Although some of these characteristics have been previously postulated or observed individually, we report them together here in aggregate via direct comparisons of cooccurring free-living and sinking-particle-attached microbial genomes from the open ocean.

## INTRODUCTION

In the ocean, heterotrophic prokaryotes play a significant role in remineralizing and transforming large fractions of marine organic matter derived from phytoplankton biomass (1, 2). This microbially transformed carbon can be released as CO_2_ into the atmosphere, transformed into dissolved organic carbon (which constitutes one of the largest actively cycled carbon reservoirs [3]), or incorporated into particulate organic carbon (POC) of living cells and decaying particulate organic matter (POM) (4). While most of this organic material is consumed in the photic zone, sinking particles that do reach abyssal depths comprise an important component of the biological pump and also provide essential nutrients and energy to the deep-sea ecosystem (5).

Particle-associated (PA) microbes may play crucial roles in POM degradation, in part due to their potential for producing extracellular enzymes that hydrolyze POC and dissolved organic matter (DOM) to lower-molecular-weight nutrients prior to cellular uptake (6, 7). These extracellular enzymes may also benefit other microbes in the PA community that lack extracellular enzymes by supplying them with lower-molecular-weight carbon sources (8, 9). Thus, sinking particles may provide a unique environmental condition with higher extracellular enzyme activity, biogeochemical transformation rates, bacterial cell densities, and overall microbial activity, in contrast to the free-living (FL) environments (10–13), leading to their contrasting microbial compositions (14, 15). However, PA communities are also highly diverse and heterogenous and may be strongly influenced by sampling regime and particle types (16–21), residence times (22), geographic location (23), depths, and seasonality (20, 24, 25). Consequently, the nature and composition of sinking particles remain to be fully characterized. Additionally, a deeper understanding of microbially driven organic carbon transformations is hampered by the complexity of processes involved in organic matter degradation as well as the challenges of linking specific microbial lineages involved with the relevant molecular processes.

Early studies contrasting coexisting PA and FL communities focused on 16S rRNA gene cloning and sequencing approaches to differentiate communities in these different habitats (14, 26). More recently, PCR-based amplicon sequencing (17, 21, 27–33), metagenomic analyses using gene-centric approaches (16, 18, 20, 28, 34–38), and examination of single amplified genomes (SAGs) have also been utilized (35). Different sampling approaches for assessing PA microbial communities have centered on collecting suspended, filter-fractionated particles (16, 28, 30, 32, 37–39) and direct collection in syringes *in situ* by divers (14) or via trapping of sinking particles by sediment traps (17, 18–21, 33–35). With respect to FL marine prokaryote communities, many metagenome-assembled genomes (MAGs) have also been recovered from the *Tara* Oceans and *Malaspina* sampling campaigns and other studies, spanning many samples and phyla (20, 40–42).

To further explore genomic differences that may exist between sinking-particle-associated (SPA) and FL microbes found throughout the water column in the oligotrophic Pacific Ocean, we leveraged time series metagenomic samples recovered at Station ALOHA in the North Pacific Subtropical Gyre, generating a total of 407 mid- to high-quality MAGs from both sample types. FL microbes (captured on 0.2-μm-pore-size filters) were collected from surface waters to a depth of 4,000 m at Station ALOHA, and metagenomes were generated, assembled, and analyzed (43–45). In parallel, 69 metagenomes collected from SPA microbes collected in sediment traps at 150 m and 4,000 m were similarly investigated. Comparative genome analysis of FL and SPA prokaryotes throughout the water column was conducted to determine microbial community composition, metabolic potential, and genomic characteristics. Our results reveal habitat-specific genomic traits that differentiate SPA from FL prokaryotes from surface waters to the abyss that relate to their organic matter utilization, metabolic lifestyles, and genomic and proteomic properties. The observed niche partitioning and habitat-specific genomic and proteomic trends identified differential adaptive characteristics and metabolic potential of SPA versus FL microbes and further illustrate their various roles in the biogeochemical transformation and organic carbon transformations in the ocean.

## RESULTS

### Recovery of MAGs from free-living and particle-associated bacteria and archaea.

To expand the genomic data from sympatric SPA and FL prokaryote communities throughout the water column, metagenomic data sets were prepared from time series samples collected from the surface to 4,000 m during Station ALOHA time series efforts and expeditions (43–47). These included 63 metagenomic samples from a time series analysis of SPA microbes reaching 4,000 m at Station ALOHA from May 2014 to November 2016 (18, 20), six metagenomic samples collected from SPA microbes at 150 m at Station ALOHA from July to August in 2015, and Hawaii Ocean time series samples collected at depths between 5 m and 4,000 m from 2014 to 2017 (43–46) (see Table S1 in the supplemental material). A total of ~5.5 Tb of metagenomic sequence data was generated and used in the MAG assemblies. Each depth- and habitat-specific metagenome sample was assembled individually, and metagenomic binning was conducted to recover metagenome-assembled genomes (MAGs). In addition, quality control was performed to remove low-quality or redundant genomes, yielding a total of 407 medium- to high-quality MAGs (each with more than 70% completeness and less than 10% contamination) (see Materials and Methods) (Table S1).

10.1128/mbio.01569-22.6 TABLE S1 Sample descriptions and metadata of metagenomes. Download Table S1, XLSX file, 0.03 MB. Copyright © 2022 Leu et al. 2022 Leu et al. https://creativecommons.org/licenses/by/4.0/ This content is distributed under the terms of the Creative Commons Attribution 4.0 International license.

**FIG 1:**
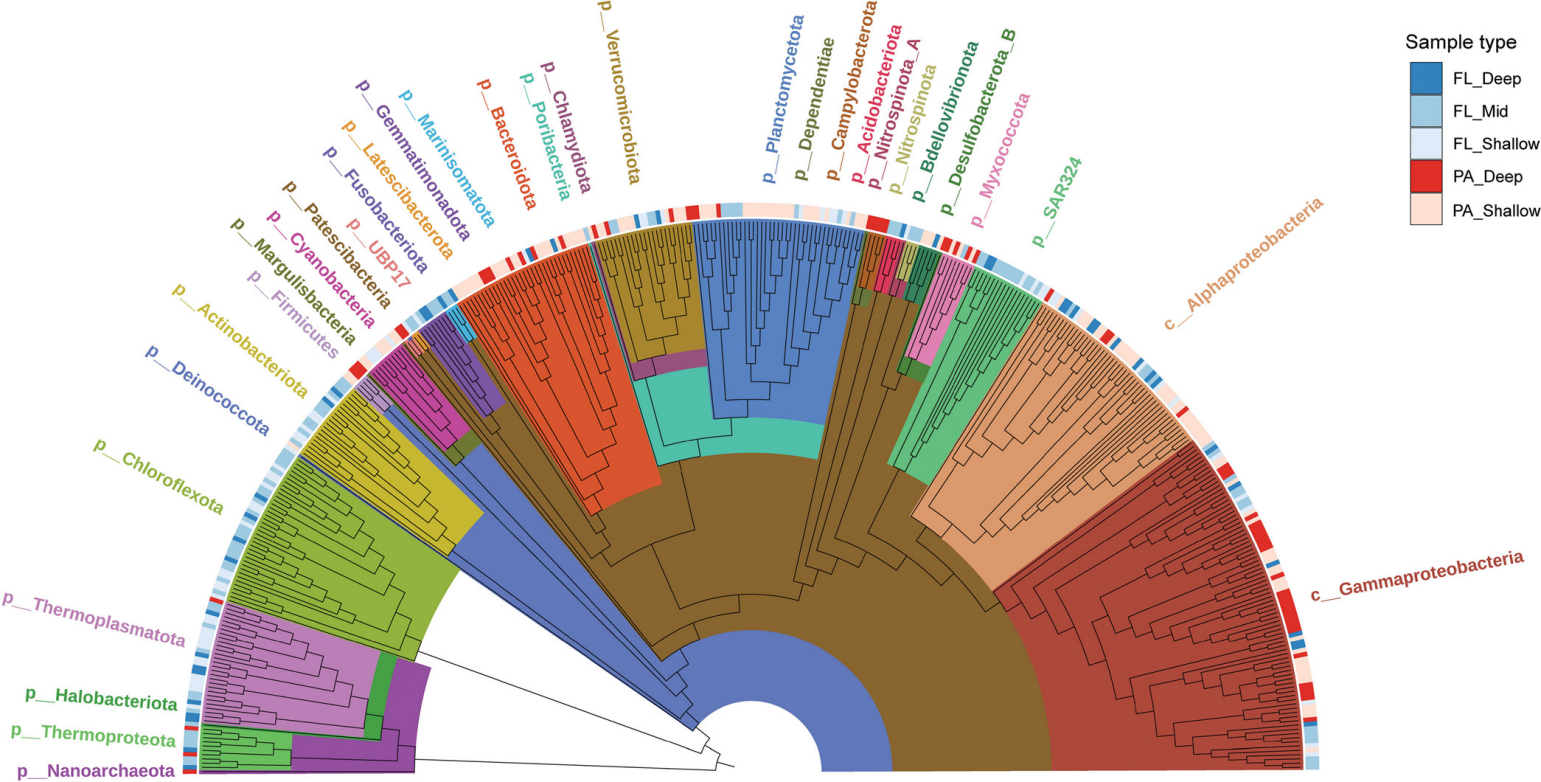
Sinking-particle-attached microbes are phylogenetically distinct from their free-living counterparts recovered from similar depths. Phylogenetic trees of the 407 dereplicated MAGs inferred using maximum likelihood analysis with a concatenated set of 122 archaeon-specific and 120 bacterium-specific marker genes. Phylum- and class-level taxonomic classifications were determined based on GTDB-Tk, GTDB v202. Each MAG was also annotated based on its sample type designation: FL, free-living; PA, particle-associated; FL_Shallow, 0 to 150 m; FL_Mid, >150 to 1,000 m; FL_Deep, 4,000 m; PA_Shallow, 150 m; PA_Deep, 4,000 m. See Table S2 in the supplemental material for more detailed taxonomic designations.

The FL and PA MAGs were classified into five sample types (Fig. 1). FL MAGs were separated into FL_Shallow, FL_Mid, and FL_Deep based on their relative abundances throughout the water column. MAGs classified as FL_Shallow were most abundant at 150 m or above, those classified as FL_Mid were most abundant from 175 m to 1,000 m, and those classified as FL_Deep were most abundant at 4,000 m. SPA MAGs that were recovered from the 150-m sediment traps in August 2015 (47) were classified as PA_Shallow. SPA Mags recovered from the 4,000-m sediment traps were classified as PA_Deep, except for those whose genomic and physiological features and distributions verify their surface water origins (20) (see Materials and Methods) (Table S2). The numbers of MAGs recovered from each of the different sample types were as follows: FL_Shallow, 64; FL_Mid, 105; FL_Deep, 43; PA_Shallow, 130; and PA_Deep, 65.

10.1128/mbio.01569-22.7 TABLE S2 Checkm statistics, taxonomic classification, and sample designation of the MAGs. Download Table S2, XLSX file, 0.04 MB. Copyright © 2022 Leu et al. 2022 Leu et al. https://creativecommons.org/licenses/by/4.0/ This content is distributed under the terms of the Creative Commons Attribution 4.0 International license.

### Phylogenetic classification of bacterial and archaeal MAGs.

To resolve the taxonomic distribution of the FL and SPA MAGs, we evaluated their placement in a reference genome tree (48, 49) (see Materials and Methods). The MAGs encompassed 28 bacterial and 4 archaeal phyla (Fig. 1; Table S2). The five most abundant bacterial phyla in our data set were *Proteobacteria*, including *Alphaproteobacteria*, *Gammaproteobacteria*, *Planctomycetota*, *Chloroflexota*, *Bacteroidota*, and *Verrucomicrobiota*. Ten other bacterial phyla were each represented by only a single MAG. This included the poorly characterized phylum UBP17, formerly known as *Cloacimonetes* (WWE1), of which only a few MAGs have been included in the genome taxonomy database to date (49). Another MAG closely related at the genus level to the UBP17 MAG recovered was the NORP72 MAG (50), which shared an average amino identity of 72.97%. This previously reported MAG was recovered from a sediment basin sample collected at ~4,484 m in the Mid-Atlantic Ridge (50), consistent with the abyssal depth origin of the UBP17 MAG reported here, which was classified as PA_Deep.

MAGs from a total of four archaeal phyla were recovered in our Station ALOHA MAG data set, which included 29 from *Thermoplasmatota*, 10 from *Thermoproteota*, 1 from *Nanoarchaeota*, and 1 from *Halobacteriota*. In contrast to the FL samples, only four archaeal MAGs were recovered from the PA communities and were from the PA_Deep sample type. With respect to genera, these MAGs included close relatives of *Nitrosopelagicus*, *Nitrosopumilus*, *Thalassarchaeum*, and *Nanoarchaeia*. While the North Pacific Subtropical Gyre (NPSG) FL and PA MAGs shared some taxa at higher classification levels, only nine genera were found in both the FL and PA data sets. These included MAGs closely related to *Alcanivorax*, *Prochlorococcus*_A, *Thalassarchaeum*, *Henriciella*, *Idiomarina*, *Nitrosopumilus*, *Nitrosopelagicus*, *Roseibacillus*_B, and *Bythopirellula* (Table S2). These data support and extend previous observations suggesting that microorganisms found in PA versus FL communities are phylogenetically distinct (14, 16, 18, 20, 26, 35, 36, 51).

### Functional genes differentiate environmental sample types.

Metabolic reconstruction was conducted to identify functional differences between the PA and FL MAGs. Protein coding sequences were annotated with KEGG, generating 9,351 unique KEGG Orthology (KO) identifiers (IDs) and clustered into 49,146 orthologous protein families. Nonmetric multidimensional scaling (NMDS) profiles based on the presence of KO IDs (Fig. S1A) and orthologous protein families (Fig. S1B) present in each MAG were generated. Globally, both the KO and orthologous protein family NMDS profiles showed strong clustering of the MAGs according to their phylum-level composition.

10.1128/mbio.01569-22.1 FIG S1 Nonmetric multidimensional scaling (NMDS) plots of the recovered MAGs based on gene annotations. (A) NMDS plot generated based on the presence/absence of KEGG Orthology (KO) annotations. (B) NMDS plot generated based on the presence/absence of orthologous protein families. Shapes indicate the sample type designation of the MAG. Color indicates the taxonomic classification of the MAG. PA and FL designations are as described in the legend to Fig. 1. Download FIG S1, PDF file, 1.1 MB. Copyright © 2022 Leu et al. 2022 Leu et al. https://creativecommons.org/licenses/by/4.0/ This content is distributed under the terms of the Creative Commons Attribution 4.0 International license.

To identify genes that were significantly associated with a given sample type, we used Scoary (52), a stringent, combined statistical test that leverages the presence/absence profiles of KO IDs and orthologous protein families (52). Overall, the PA_Shallow and PA_Deep samples showed greater numbers of genes that were significantly enriched than the FL sample types (false discovery rate-corrected *P* value < 0.05) (Fig. S2A and B and Tables S3A, S3B, and S4). The PA_Shallow and PA_Deep MAGs shared a large subset of significantly enriched genes in terms of both KO and orthologous protein family annotation (545 and 290, respectively) (Fig. S2A and B). For FL samples, only FL_Shallow and FL_Mid showed some gene overlap. No overlap of significantly enriched genes was found between FL and PA samples in terms of orthologous protein families. However, four KO IDs were shared between FL_Shallow and PA_Shallow that encoded zinc/manganese transport system ATP-binding protein as well as deoxyribodipyrimidine photolyase, bacteriorhodopsin, and phytoene desaturase (involved in carotenoid synthesis). Most of these genes are involved in light-driven energy generation or DNA damage repair, consistent with their surface origins. Two KO IDs were found shared between FL_Deep and PA_Deep that encoded the mercuric ion transport protein (merT) and cobalt-zinc-cadmium efflux system protein (czcD), suggesting potentially higher concentrations or bioaccumulation of these cationic metals at greater depths.

10.1128/mbio.01569-22.2 FIG S2 Venn diagram of significantly enriched genes. (A and B) Venn diagrams of significantly enriched genes based on KEGG Orthology (KO) annotations and orthologous protein family. Significantly enriched KOs and orthologous gene families were determined using Scoary with a *q* value of <0.05 for Fisher’s exact test. Download FIG S2, PDF file, 0.5 MB. Copyright © 2022 Leu et al. 2022 Leu et al. https://creativecommons.org/licenses/by/4.0/ This content is distributed under the terms of the Creative Commons Attribution 4.0 International license.

10.1128/mbio.01569-22.8 TABLE S3 Scoary enrichment of KO IDs (A) and orthologous protein families (B) based on sample types. Download Table S3, XLSX file, 0.4 MB. Copyright © 2022 Leu et al. 2022 Leu et al. https://creativecommons.org/licenses/by/4.0/ This content is distributed under the terms of the Creative Commons Attribution 4.0 International license.

10.1128/mbio.01569-22.9 TABLE S4 Count tables of transporter genes (A), peptidase genes (B), CAZyme genes (C), and extracellular secretion system genes (D) included in MAGs. Download Table S4, XLSX file, 0.1 MB. Copyright © 2022 Leu et al. 2022 Leu et al. https://creativecommons.org/licenses/by/4.0/ This content is distributed under the terms of the Creative Commons Attribution 4.0 International license.

The most enriched KO IDs identified by Scoary were significantly associated with the PA_Shallow MAGs (Table S3A and B). These genes encoded dihydrolipoamide dehydrogenase (DLD), i.e., an oxidoreductase linked to central metabolism, thioredoxin, which is involved in defending against oxidative stress, 1-acyl-sn-glycerol-3-phosphate acyltransferase, which is involved in glycerolipid metabolism, and most prevalent of all, a large family of proteins categorized as putative ABC transporters (Table S3A and B).

### Substrate uptake capabilities of FL versus PA bacteria and archaea.

Previous studies have shown that transporters of carbohydrates and energy sources are ubiquitous in heterotrophic prokaryotic communities throughout the open water column, from the surface to abyssal depths, and are crucial for organic matter transformation (53). To investigate essential nutrient uptake capabilities, transporters with compound specificity were examined in the different sample types (Fig. S3 and Table S4A). In general, MAGs in the SPA samples (both surface and deep) encoded a higher diversity of transporters than FL MAGs. Genes encoding transporters with substrate affinity to amino acids, aromatic compounds, carbohydrates, metal, nitrate/nitrite, peptide, phosphate, and spermidine/putrescine were more highly represented in SPA MAGs than FL MAGs. The PA_Shallow group had a relatively greater representation of MAGs (9.2%) that included genes for taurine transporters than did the PA_Deep group (1.5%) and the FL sample types (0 to 2.9%). A large percentage of MAGs in all sample types included genes for the assimilatory sulfate reduction pathway. Six percent of the PA_Shallow MAGs contained sulfate/thiosulfate transporter genes that were not identified in other sample types. The absence of the sulfate/thiosulfate transporters in some SPA microbes may indicate their reliance instead on reduced sulfur compounds, thereby alleviating energetic demands for transport and assimilatory reduction of sulfate (54). The PA_Deep group had the greatest relative proportion of MAGs encoding amino acids, aromatic compounds, di-oligopeptides, nitrate/nitrite, peptide, and spermidine/putrescine transporters in comparison to PA_Shallow and all FL MAG groups (Fig. S3; Table S4A), suggesting that deep-sea SPA microbes may be better adapted to incorporating these substrates at greater depths.

10.1128/mbio.01569-22.3 FIG S3 Counts of MAGs encoding substrate-specific transporters. Genes encoding putative transporters were grouped based on their substrate specificity. Download FIG S3, PDF file, 0.1 MB. Copyright © 2022 Leu et al. 2022 Leu et al. https://creativecommons.org/licenses/by/4.0/ This content is distributed under the terms of the Creative Commons Attribution 4.0 International license.

10.1128/mbio.01569-22.4 FIG S4 Counts of MAGs encoding substrate-specific CAZymes. Genes encoding putative CAZymes were annotated against dbCAN (3.0) with default parameters (107) and filtered to retain hits with E values of <1e−102 and an HMM coverage of ≤0.35. Filtered genes were further classified to their subfamily level, and their EC numbers were determined using eCAMI. Download FIG S4, PDF file, 0.1 MB. Copyright © 2022 Leu et al. 2022 Leu et al. https://creativecommons.org/licenses/by/4.0/ This content is distributed under the terms of the Creative Commons Attribution 4.0 International license.

A greater proportion of MAGs encoding mannopine transporters were identified in the FL samples than in the PA samples (~6.2% versus ~1.2%) (Fig. S3; Table S4A). The substrate mannopine and other opine-like compounds are secondary amine derivatives that are utilized by many different bacteria as carbon, energy, and nitrogen sources (55). While opines are commonly found in crown gall tumor tissues in plants, other potential sources of opines have been isolated from marine invertebrates, such as mollusks, cnidarians, and sponges (56). This suggests that the mannopine-like transporters in FL_MAGs may acquire dissolved mannopine (or other opine-like substrates) derived from marine invertebrates or eukaryotic sources.

### Polymer-degrading enzymes of PA versus FL bacteria and archaea.

**FIG 2:**
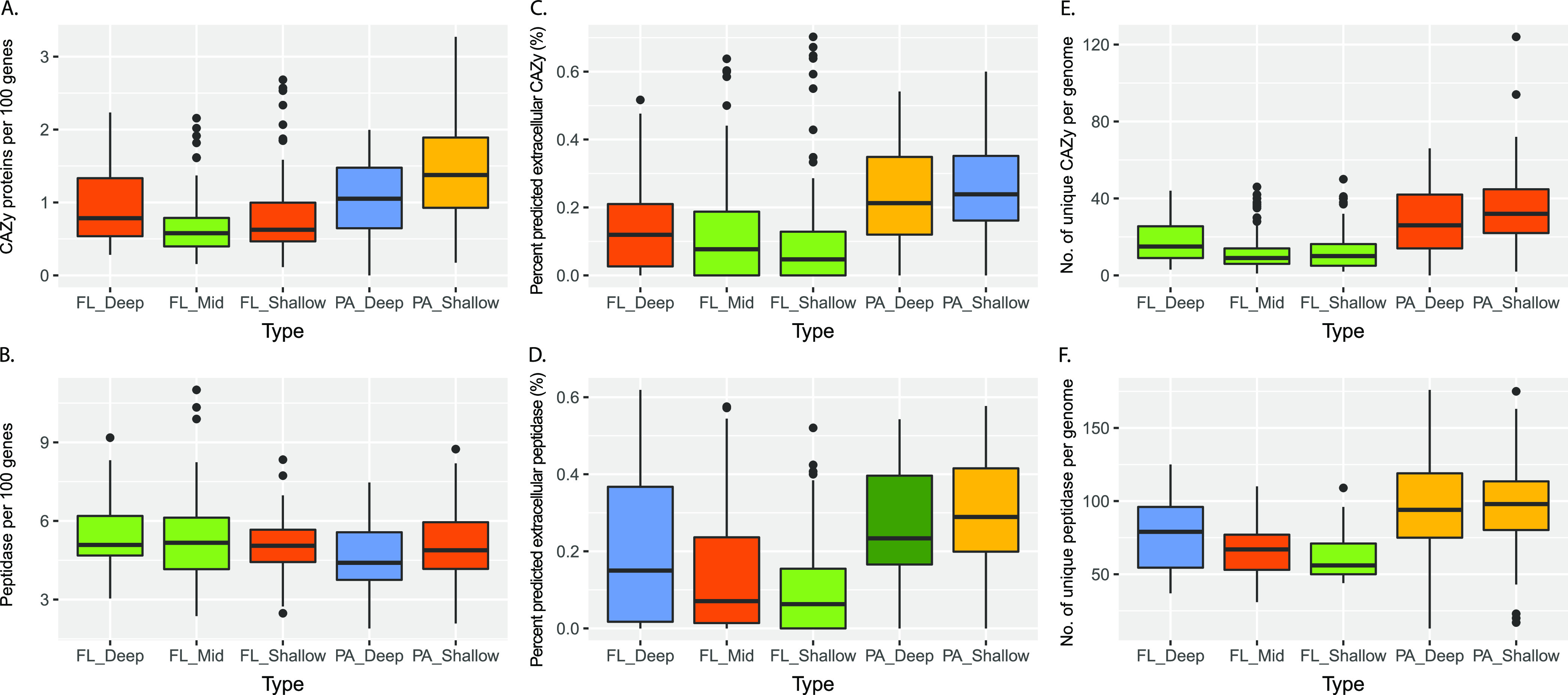
Sinking-particle-attached microbes contain relatively more secretory CAZyme and peptidase genes than do their free-living counterparts. (A and B) Counts of genes encoding CAZymes (A) and peptidases (B) per 100 genes in MAGs. (C and D) Percentages of genes encoding extracellular CAZymes and extracellular peptidases (D) in MAGs. (E and F) Numbers of unique CAZymes (E) and peptidases (F) per genome. Shared color denotes no significant difference. Box plots show medians and enclose 1st and 3rd quartiles. Whiskers denote 1.5 times the interquartile range (IQR) of the lower and upper quartiles. Statistics are based on the Tukey-Kramer test, and different colors denote significant differences (*P < *0.05). PA, particle-associated.; FL, free-living; FL_Shallow, 0 to 150 m; FL_Mid, >150 to 1,000 m; FL_Deep, 4,000 m; PA_Shallow, 150 m; PA_Deep, 4,000 m.

Given the higher prevalence and diversity of transporters in SPA MAGs, POC solubilization capabilities of both SPA and FL MAGs were investigated. Extracellular hydrolytic enzymes that include peptidases and carbohydrate-active enzyme (CAZymes), key components in particulate organic carbon hydrolysis, were examined in terms of their abundance, diversity, and function (Fig. 2; Table S4B and C).

The SPA MAGs possessed a significantly higher count of genes encoding peptidases and CAZymes per genome than the FL MAGs (Fig. 2A and B). A similar trend could also be observed for genes encoding secretory peptidases and CAZymes, with SPA MAGs showing higher average percentages of these secreted extracellular enzymes than FL MAGs (Fig. 2C and D). A significantly higher percentage of secretory peptidases were also observed in FL_Deep MAGs than the other FL sample types. In terms of functional diversity, the PA MAGs showed the highest overall diversity of peptidase and CAZyme classes per genome in comparison to the FL MAGs (Fig. 2E and F). Within the FL MAGs, the FL_Deep MAGs had a higher diversity of peptidase classes than MAGs of other depths and a CAZyme diversity similar to that of FL_Shallow MAGs.

### Polysaccharide degradation potential in SPA versus FL bacteria and archaea.

**FIG 3:**
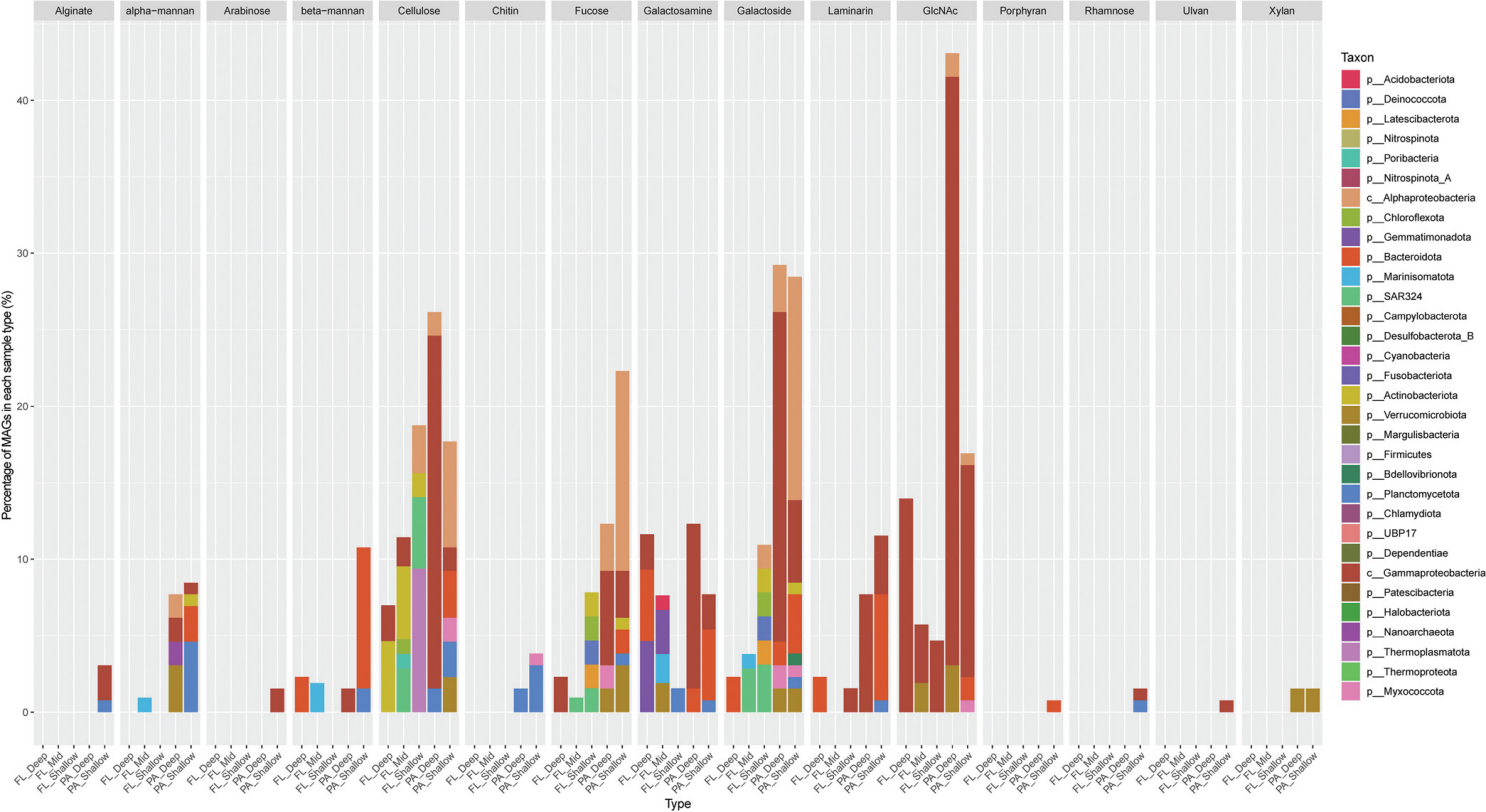
Sinking-particle-attached microbes contain relatively more polysaccharide-degrading genes than do their free-living counterparts. Percentages of genomes encoding substrate-specific CAZymes (see Table S4B in the supplemental material), separated by sample types. MAGs are colored based on their phylum- or class-level taxonomic classification. PA, particle-associated; FL, free-living; FL_Shallow, 0 to 150 m; FL_Mid, >150 to 1,000 m; FL_Deep, 4,000 m; PA_Shallow, 150 m; PA_Deep, 4,000 m.

Since polysaccharides constitute a large fraction of DOC (57) and POC (58), identifying genes encoding CAZymes and their substrate specificity in MAGs is crucial for determining the putative carbon source as well as the specific microbial members driving organic carbon transformation. Putative CAZymes with fully defined EC numbers (complete with four identifiers) were used to identify specific genes encoding glycoside hydrolase (GH) and polysaccharide lyase (PL) and the putative polysaccharides they target. In total, 45 EC numbers were identified that correspond to potential hydrolysis of 34 different putative substrates, which included common carbohydrates (e.g., starch) as well as marine relevant polysaccharides such as laminarin, xylan, and chitin (7, 59–61). PA_Shallow possessed greater diversity and a higher percentage of microbes capable of hydrolyzing a large variety of polysaccharides, followed by PA_Deep and the FL samples (Fig. 3; Fig. S4 and Table S4B).

Only one CAZyme, α-l-arabinofuranosidase (GH51), was found to be enriched in FL MAGs in comparison to PA MAGs (Table S4B). This enzyme is linked to the cleavage of α-arabinofuranoside from hemicellulose (62). In contrast, a large number of biochemically well characterized CAZymes (63–65) were present only in SPA MAGs, including β-xylosidases (GH52), ulvan lyase (PL24 and PL25), rhamnogalacturonan α-l-rhamnohydrolase (GH106), α-l-rhamnosidase (GH106), alginate lyase (PL6 and -14), endo-α-1,5-l-arabinanase (GH43), β-l-arabinofuranosidase (GH146), chitinase (GH18), and β-porphyranase (GH16). These CAZymes hydrolyze polysaccharides such as alginate, porphyran, ulvan, and xylan, which are structural components of algae, and chitin, a highly abundant polysaccharide in marine environments. These CAZymes also hydrolyze an assortment of polysaccharides, releasing sugars such as rhamnose, xylan, and arabinose, which are found enriched in spring phytoplankton blooms (66).

Other marine relevant CAZymes that are enriched in SPA MAGs also include α-mannosidase (GH38 and GH92), β-mannosidase (GH2), α-l-fucosidase (GH29 and GH151), β-glucosidase (GH1, -2, and -3), cellulase (GH5, -6, and -45), cellobionic acid phosphorylase (GH94), α-*N*-acetylgalactosaminidase (GH109 and -114), β-galactosidase (GH2 and -42), and α-galactosidase (GH4, -31, -36, and -97). The enrichment of these CAZymes in SPA MAGs also correlates well with the spike in multiple sugars detected during summer phytoplankton blooms, such as mannan, fucose, glucose, galactosamine, and galactose (67).

The most enriched CAZymes identified in any sample type were in the PA_Deep sample, where 43.1% of all MAGs contained genes for β-*N*-acetylhexosaminidase (GH3, -20, -84), which is involved in the hydrolysis of N*-*acetylated oligo/polysaccharides such as chitooligosaccharides and chitin, which are prevalent and abundant in marine crustaceans. MAGs containing genes for β-*N*-acetylhexosaminidase were present only within the *Gammaproteobacteria* and include the orders *Arenicellales*, *Burkholderiales*, *Enterobacterales*, *Nitrosococcales*, *Pseudomonadales*, UBA11654, and *Xanthomonadales*. Lastly, genes encoding laminarin endo-1,3-β-d-glucosidase (GH16 and -81) were found to be enriched in 25 SPA MAGs, which degrades laminarin, a polysaccharide abundant in microalgae such as diatoms (61). These SPA MAGs belonged to multiple phyla, including *Proteobacteria*, *Planctomycetota*, *Verrucomicrobiota*, and *Bacteroidota*. Most of the *Bacteroidota* MAGs belonged to the *Flavobacteriaceae* family, which are well known degraders of polysaccharides and associated with phytoplankton blooms (67). The presence of these CAZymes in the PA_Shallow and PA_Deep MAGs suggests that many of these polysaccharides are likely more available as substrates in POC and accessible as polymeric substrates for SPA microorganisms. Our observations here are consistent with a recent study (68) that suggested that particle-associated enzymatic profiles had a greater representation of peptidases and a broader spectrum of polysaccharide hydrolases than did corresponding bulk seawater samples.

### Enrichment of TCSs, extracellular secretion systems, and prophage in SPA bacteria and archaea.

Two-component sensory systems (TCSs) enable rapid transcriptional responses to environmental variations, including light, temperature, and nutrient availability (69). TCSs are composed of a response regulator protein and a histidine kinase, which when activated by a molecule or by other physical stimuli induce a conformational change and interact with the response regulator protein. This typically stimulates binding of the response regulator to a DNA promoter region, allowing transcription of the genes in the downstream operon (70). Prior studies have also indicated that a lack of two-component regulatory systems in bacterial genomes may be a hallmark of oligotrophy for bacteria (69).

**FIG 4:**
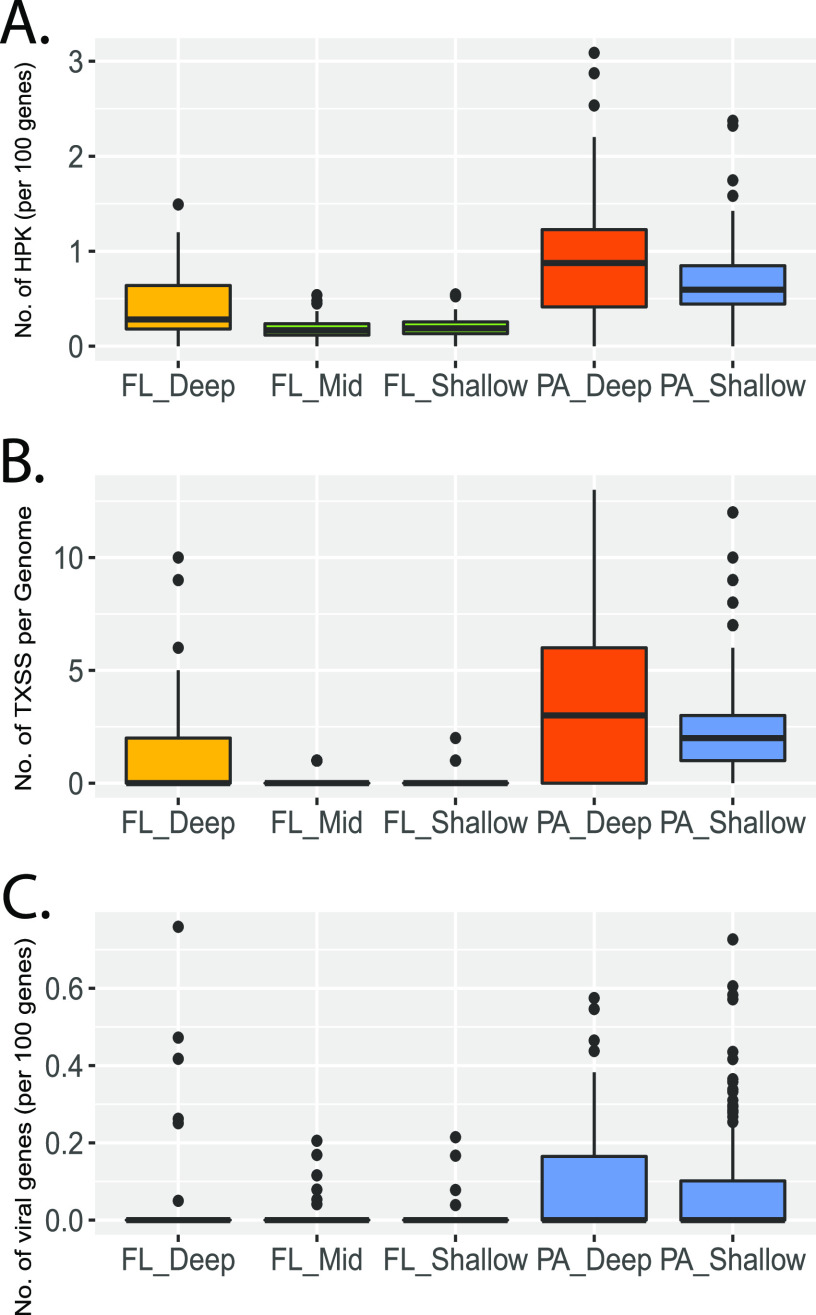
Sinking-particle-attached microbes contain more histidine kinase, extracellular secretion system, and prophage-associated genes than do their cooccurring free-living counterparts. Variance of histidine kinase (A), secretion system (B), and virus-associated (C) genes in MAGs from different sample types. Histidine kinases were identified based on hits to pfam02518, pfam13581, pfam13589, pfam14501, and pfam07536. Secretion system genes include genes encoding bacterial flagellum, T1SS, T2SS, T4P, T5aSS, T5bSS, T6SSi, T9SS, Tad(tight_adherence), and pT4SSt secretion systems. Prophage-associated gene markers included genes encoding phage CI repressor, phage capsid proteins, phage excisionase, and phage tail proteins. Box plots show medians and enclose 1st and 3rd quartiles. Whiskers denote 1.5 times the IQR of the lower and upper quartiles. Statistics are based on the Tukey-Kramer test, and different colors denote significant differences (*P < *0.05). Shared color denotes no significant difference. PA, particle-associated; FL_Shallow, 0 to 150 m; FL_Mid, >150 to 1,000 m; FL_Deep, 4,000 m; PA_Shallow, 150 m; PA_Deep, 4,000 m.

Examination of the SPA and FL MAGs for genes encoding TCSs showed significantly higher representation of histidine kinases (HPK; normalized to total gene content) in PA MAGs than in FL MAGs (Fig. 4A). In addition, PA_Deep MAGs possessed higher numbers of HPK than PA_Shallow MAGs, consistent with the higher organic carbon energy content previously documented for abyssal sinking particles (25). With respect to FL microbes, FL_Deep genomes had a higher HPK gene content than FL_Shallow and FL_Mid genomes. In conjunction with the higher percentage of secreted peptidases (Fig. 2C) found in FL_Deep MAGs, this suggests a more frequent association of deep-sea microbes with particles (36).

Extracellular protein secretion systems (ESSs) are widespread among bacteria and archaea and are central components in fimbria- and pilus-associated attachment and adhesion, nutrient transport, predation, cell-cell interactions, surface colonization, and pathogenicity in diverse environmental contexts and settings (71–74). The diversity of ESS processes can be catalogued in part by the underlying mechanism of transport for any given ESS (75). Examination of the SPA and FL microbes showed that PA_Deep and PA_Shallow were significantly enriched in ESSs, compared to FL MAGs (Fig. 4B; Fig. S5 and Table S4D). In particular, SPA microbes had greater representation of genes coding for Tad systems, as well as ESS types I, II, III, IV, V, and IX, in their genomes than FL microbes at all depths. Two exceptions, however, included type 5cSS and type 4SSt, whose representations in FL and SPA microbial genomes were generally equivalent. SPA MAGs also had a higher representation of flagella, closely followed by a similar representation in FL_Deep microbes (Fig. S5). With respect to FL MAGs, FL_Shallow and FL_Mid genomes had a lower genomic representation of ESSs than their FL_Deep counterparts (Fig. 4B; Fig. S5 and Table S4D).

10.1128/mbio.01569-22.5 FIG S5 Frequency of different extracellular secretion systems in particle-associated versus free-living bacterial genomes in the water column. Genes encoding bacterial secretion systems were predicted using MacSysFinder with the TSScan reference database (v1.0rc1). Download FIG S5, PDF file, 0.1 MB. Copyright © 2022 Leu et al. 2022 Leu et al. https://creativecommons.org/licenses/by/4.0/ This content is distributed under the terms of the Creative Commons Attribution 4.0 International license.

Prior work has suggested that deep-sea bacteria may have larger genomes than bacteria in the epipelagic zone (46, 76), and our observations here show that particle-attached bacteria on average have larger genomes than sympatric free-living counterparts. This provides potentially more genome “real estate” for expanded metabolic versatility and potentially greater capacity to accommodate mobile genetic elements. Examination of phage-associated genes in SPA versus FL MAGs revealed that SPA microbes (regardless of depth horizon) had a higher representation of phage genes, including both temperate phage markers and phage capsid structural genes (Fig. 4C). These data are consistent with the expectation that the larger genomes of particle-attached and deep-sea bacteria can accommodate greater numbers of mobile genetic elements.

### Intrinsic genomic and proteomic traits differentiate SPA from FL bacteria and archaea.

PA microbial communities are often viewed as “hot spots” for microbial activity in comparison to their FL counterparts, based on their larger cell size and cell densities, higher enzyme activities, and overall rates of heterotrophic microbial metabolism (10, 12, 13). This is a result of the aggregation of phytodetrital matter, fecal pellets, and high-molecular-weight nutrient-rich organic material such as proteins and polysaccharides, which are consumed by heterotrophic bacteria (77–79). Thus, nutrient-replete microhabitats on particles may influence the carbon and nitrogen budgets and core genomic properties (44, 80–83).

**FIG 5:**
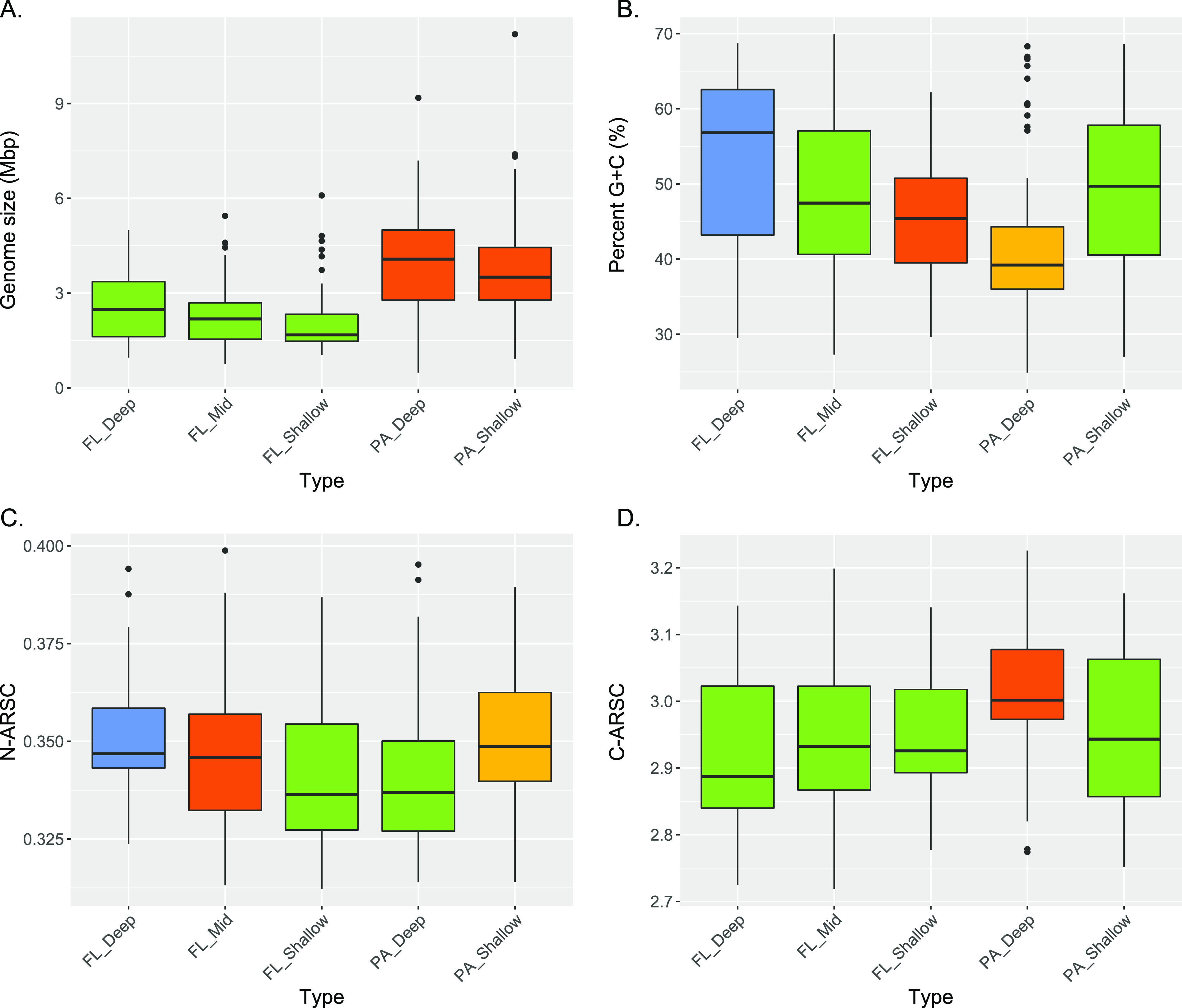
Sinking-particle-attached microbes have different genomic and proteomic compositions than their free-living counterparts. Variances of genome size (A), GC content (B), N-ARSC (C), and C-ARSC (D) in MAGs from the different sample types. Box plots show medians and enclose 1st and 3rd quartiles. Whiskers denote 1.5 times the IQR of the lower and upper quartiles. Statistics are based on the Tukey-Kramer test, and different colors denote significant differences (*P < *0.05). Shared color denotes no significant difference. PA, particle-associated; FL_Shallow, 0 to 150 m; FL_Mid, >150 to 1,000 m; FL_Deep, 4,000 m; PA_Shallow, 150 m; PA_Deep, 4,000 m.

To further test whether observable genomic variability in MAGs was evident across multiple sample types and depths, we examined variability in a variety of genomic traits in SPA versus FL microbes throughout the water column. Analysis of these MAG genomic traits showed that SPA prokaryotes (from both deep and shallow water) had significantly larger genome sizes on average than FL microbes (from all depths) (Fig. 5A). In terms of GC content, there was a significant increase from FL_Shallow to FL_Deep MAGs, whereas PA_Deep MAGs had a lower GC content than PA_Shallow MAGs (Fig. 5B). In the context of predicted protein elemental composition, the average number of nitrogen atoms per amino acid residue side chain (N-ARSC) generally correlates with GC content variability in cultivar genomes (83). The results for the FL MAGs were consistent with recent gene-centric analyses showing increases in both GC content and N-ARSC values that tracked *in situ* inorganic nitrogen availability and depth for FL bacterioplankton at Station ALOHA (Fig. 5C) (44). Notably, the PA MAGs showed a depth trend opposite to that of FL MAGs, with PA_Deep MAGs having lower GC and N-ARSC values than PA_Shallow MAGs (Fig. 5C). As for the average number of carbon atoms per amino acid residue side chain (C-ARSC), only the PA_Deep MAGs possessed statistically higher values than all other sample types (Fig. 5D). These genomic trends in SPA bacteria may be driven by the elemental stoichiometry of organic carbon in shallow versus deep sinking particles, since shallow-water particles trend toward a higher organic nitrogen content, whereas sinking particles reaching the abyss are relatively depleted in organic nitrogen (25).

### Growth efficiency estimates for PA versus FL bacteria and archaea.

**FIG 6:**
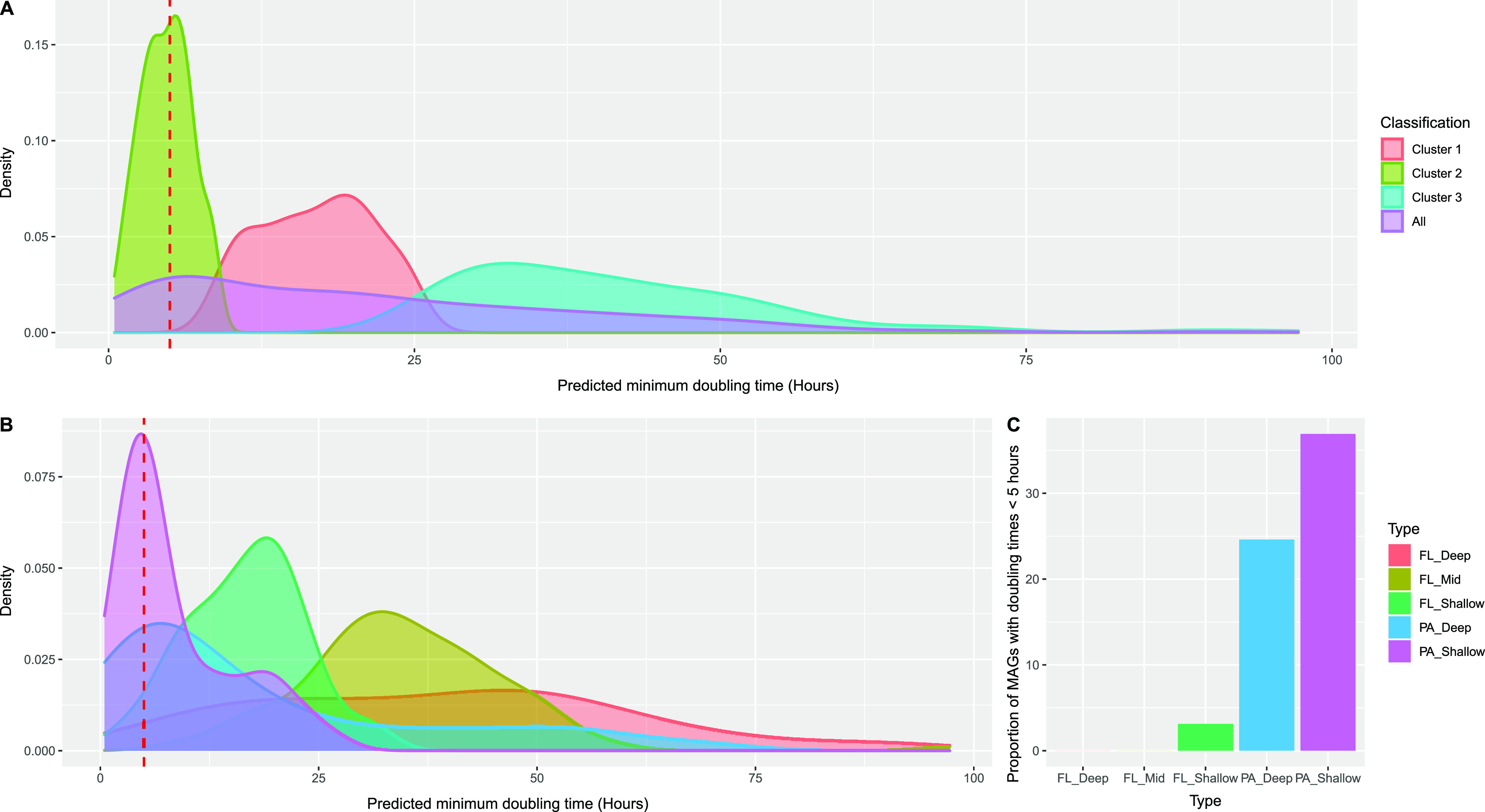
Predicted growth efficiencies of sinking-particle-attached microbes are greater than those of free-living microbes. (A) Predicted temperature-corrected maximum growth rates of all recovered MAGs, clustered based on a Gaussian mixture model. (B) Predicted temperature-corrected maximum growth rates of all recovered MAGs, clustered based on their sample types. (C) Proportion of MAGs in each sample type with temperature-corrected predicted doubling times of <5 h. A vertical dashed red line represents a doubling time of 5 h. FL, free-living; PA, particle-associated; FL_Shallow, 0 to 150 m; FL_Mid, >150 to 1,000 m; FL_Deep, 4,000 m; PA_Shallow, 150 m; PA_Deep, 4,000 m.

Recently, codon usage bias (CUB) metrics in genes encoding ribosomal proteins have been used to estimate the maximum growth rates (minimum doubling times) of microorganisms in both laboratory and environmental settings (84–87). We therefore tested whether CUB metrics might differentiate between the different SPA and FL sample types and genomes (Fig. 6; Table S5). Using a Gaussian mixture model, the distribution of the SPA and FL MAGs separated into three clusters with predicted mean minimum doubling times of 4.7, 16.9, and 36.7 h, respectively (Fig. 6A). These three clusters did not correspond to depth of origin, even though they spanned a range of 4,000 m, with pressure differences of >400 atm and temperature differences of >22°C. Instead, the three clusters were strongly associated with PA_Shallow and PA_Deep in one cluster, and the FL samples split between two clusters. Specifically, the majority of PA_Deep and PA_Shallow MAGs had greater predicted growth efficiencies (as estimated by CUB-based theoretical maximum growth rates) than did the FL MAGs (Fig. 6B and C). While individual MAGs may differ in part due to phylogenetic trends, the overall higher predicted growth efficiencies among PA prokaryotes than among FL prokaryotes generally supports the postulated copiotrophic lifestyles of SPA microbes compared to free-living oligotrophs throughout the water column.

10.1128/mbio.01569-22.10 TABLE S5 Estimated growth efficiencies based on MAG codon usage biases. Download Table S5, XLSX file, 0.03 MB. Copyright © 2022 Leu et al. 2022 Leu et al. https://creativecommons.org/licenses/by/4.0/ This content is distributed under the terms of the Creative Commons Attribution 4.0 International license.

## DISCUSSION

The application of phylogenetic, genomic, and metagenomic approaches to the study of particle-attached versus free-living microbes is now providing new insights into microhabitat dynamics throughout the water column, as well as identifying habitat-specific adaptive characteristics. We report here microbial genomes associated with sediment trap-collected sinking particles versus filtered free-living microbes from the photic zone to the abyss, to identify any habitat-specific genomic traits. The majority of the SPA MAGs were found to be phylogenetically unrelated to FL MAGs. Only a few genera were captured in both the FL and PA samples and included phototrophic species like *Prochlorococcus* as well as motile heterotrophic copiotrophs like *Idiomarina*. These results further illustrate the distinctive microhabitat-specific phylogenetic, physiological, and ecological divergence between SPA and FL microbial communities (14, 16, 18, 20, 26, 35, 36, 51).

Rapidly sinking particles are rich in nutrients (including polysaccharides and proteins) and energy (7, 25, 61, 67). Consequently, we observed a higher percentage of secreted peptidases and CAZymes in SPA microbes than in FL microbes. This observation is consistent with the prediction that microbes inhabiting diffusion-limited environments (e.g., soils, sludges, sediments) encode more extracellular enzymes, in part because they diffuse away more slowly in structured (particles) versus unstructured (bulk seawater) habitats. SPA MAGs were also found to be enriched in CAZymes that degrade different polysaccharides known to be abundant in marine habitats. Notably, those microbes capable of hydrolyzing laminarin and known to be abundant in marine environments (7, 61, 67) were most highly represented in the SPA samples. With predictions of laminarin accounting for up to 50% of POC in sinking diatom-containing particles (61), the data suggest that SPA microbes may have a considerable influence on laminarin degradation and associated carbon export to the abyssal depths in the open ocean.

Previous metagenomic and metaproteomic studies of 0.2-μm-filtered samples throughout the water column reported that total peptidases and CAZymes decreased from epipelagic (0 to 200 m) to bathypelagic (>1,000 m) microbial communities, while the percentage of extracellular enzymes and their diversity increased with depth (36). In our genomic survey, a decrease in total peptidase and CAZyme genes in genomes with depth was observed only in the SPA MAGs. However, the percentage of extracellular peptidases and CAZymes was not significantly higher with depth in SPA or FL sample types. Methodological differences in particle sampling methods (e.g., hand collection by SCUBA divers versus serial filtration versus sediment traps), along with differences in spatial resolution, analytes, and analytical strategies, may in part explain the differences between our results and prior studies (36). Our CAZyme, histidine kinase, and secretion system results do, however, support the postulate that filter-collected, suspended bathypelagic prokaryotes appear more adapted to particle colonization than their epipelagic counterparts (36). Additionally, the enrichment of total and secretory hydrolytic extracellular enzymes and their elevated diversity in SPA microbes suggest their greater metabolic potential for solubilization of a wide variety of POC substrates in sinking particles. Overall, the higher diversity of transporters in SPA MAGs also suggests that efficient solubilization may produce a larger variety of nutrients which are subsequently taken up by particle-associated communities.

Given their particle-associated lifestyles, we postulated that SPA microbial genomes are also enriched in genes that promote environmental sensing and response, motility, attachment, colonization, and cell-cell interactions. Consistent with this hypothesis, both the shallow- and deep-water SPA microbes contained greater proportions of genes associated with two-component regulatory systems, extracellular secretion systems, and flagella than did FL microbes from the same depth horizons. The genomic distributions of TCSs support prior observations with respect to general metabolic types (69) and suggest a greater potential for environmental sensing and response capacity in SPA microbes than in their sympatric oligotrophic FL counterparts. The diverse array and elevated levels of ESS genes in SPA microbes compared to FL microbes also support their postulated greater capacity for attachment, motility, and cell-cell interactions. Additionally, the elevated levels of phage and prophage marker genes in SPA versus FL microbial genomes are consistent with hypotheses suggesting that nutrient-replete, high-cell-density habitats may favor retention of greater numbers of lysogens and prophage (85). Taken together, the greater representation of TCS, ESS, and phage genes in SPA microbes likely contributes to their expanded behavioral, regulatory, and metabolic traits, reflecting adaptations to heterogeneous and ephemeral microhabitats.

Prior gene-centric studies have demonstrated that genome size, GC content, and genome and proteome nitrogen content tend to positively correlate with environmental inorganic nitrogen availability among FL bacterioplankton populations (44, 81, 82). The genome-based analyses reported here generally recapitulate these trends for FL bacterioplankton, with increased genome size, GC content, and N-ARSC being a hallmark for deeper-water FL genomes, in part driven by increased dissolved inorganic nitrogen availability.

SPA microbes, however, showed an opposite trend with respect to DNA GC content and proteome N-ARSC with increasing depth. Strikingly, shallow-water SPA microbes collected at 150 m had higher DNA GC and proteome nitrogen contents than did deeper, 4,000-m SPA microbes. The higher genome and proteome nitrogen content in shallow- versus deep-water SPA microbes is consistent with recent studies of the elemental composition and energy content (in Joules per unit mass of organic carbon) (25) of sinking particles. Specifically, sinking particles collected at 4,000 m had relatively higher organic carbon-to-nitrogen (C:N) contents (C:N, ~10:1) than did sinking particles collected at 150 m (C:N, ~6:1) (25). In total, our new data suggest that resource limitation may also drive genome and proteome elemental stoichiometry in SPA microbes. Specifically, more nitrogen-depleted particles found at greater depths appear to select for SPA microbes having lower DNA GC content and less nitrogen in their genomes and proteomes than their shallow-water, more nitrogen-enriched SPA counterparts. These trends suggest that shallow-water SPA microbes may rely more on particle-derived organic nitrogen compounds than on more oxidized bulk inorganic nitrogen sources (NO_3_^−^, NO_2_^−^) to reduce the energetic costs of nitrogen acquisition. Our data further suggest that as particles sink and nitrogen depletion ensues, SPA microbial assemblages undergo dynamic compositional shifts in response to the more nutrient-depleted particle microenvironments found at greater depths.

Codon usage bias has been suggested to be capable of determining the *in situ* maximum growth rates (minimum doubling times) of prokaryote environmental settings (84–87). Accurate *in situ* growth rates cannot necessarily be determined by this approach, however, since temperature, pressure, nutrient availability, competition, mortality, and epigenetic phenomena are all expected to exert profound effects on actual *in situ* microbial growth rates in nature. Nonetheless, this approach may provide a useful metric for comparing relative growth efficiencies of different bacteria and archaea from similar habitats. We observed here that SPA and FL MAGs partitioned specifically into discrete categories based on codon usage bias estimates that generally reflected their copiotrophic versus oligotrophic lifestyles, respectively. This further reflects the major influence across phyla of nutrient availability as a central ecological and evolutionary driver of both SPA and FL gene acquisition, genome evolution, and metabolic capabilities.

In total, this genome-centric study demonstrates for the first time the aggregate genomic features and gene repertoires that differentiate sympatric SPA from FL bacteria and archaea in the open ocean and define their phylogenetic metabolic and behavioral characteristics. These SPA and FL genomic traits reflect both the physicochemical structure and chemical composition of their environment as well as their competitive and synergistic interactions throughout the water column. Future directed efforts exploring the biological properties and biogeochemical activities of SPA and FL microbes *in situ* at greater spatiotemporal resolution may help to provide a better understanding of the complex mechanisms of POM and DOM cycling, which in part drive the ocean’s biological carbon pump.

## MATERIALS AND METHODS

### Sample collection.

Metagenomic data for the PA_Shallow samples were generated from DNA extracted from bulk sinking particles collected in net traps at 150 m as described by Farnelid et al. (47). Briefly, particles collected in the traps were filtered through a 0.2-μm-pore-size Supor filter (Pall Corporation, New York, NY, USA), and DNA was subsequently extracted using a Qiagen plant minikit and a QIAcube (Qiagen, Valencia, CA, USA).

Metagenomic data from the PA_Deep samples were derived from the 63 samples collected from the deep-sea sequencing sediment traps at 4,000 m, as previously described (18, 20). Briefly, particles collected in preservative-containing trap cups were centrifuged to collect the sinking particulate material, and DNA was subsequently extracted using a Qiagen DNeasy PowerBiofilm kit (Qiagen, Valencia, CA, USA).

Metagenomic sequence data from FL samples (see Table S1 in the supplemental material) were derived from Station ALOHA time series samples collected between depths of 5 and 4,000 m on Hawaii Ocean Time series cruises. Samples were collected, processed, and DNA extracted as previously described (44–46). Briefly, 2 to 4 L of seawater was collected using conductivity-temperature-depth (CTD)-attached Niskin bottles and filtered (with no prefiltration) onto a 0.2-μm-pore-size, 25-mm Supor filter (VWR 28147-956). After filtration, the filters were removed and stored in 300 μL of RNAlater (Thermo Fisher Scientific, Waltham, MA) at −80°C. DNA extractions were performed by thawing filters on ice, removing the RNAlater, and adding 400 μL of sucrose lysis buffer (final concentrations of 40 mM EDTA, 50 mM Tris (pH 8.3), and 0.75 M sucrose). Cell homogenization was performed using a TissueLyser (Qiagen, Germantown, MD) programmed at 30 Hz for two rounds of 1 min each. This was followed by the addition of 0.5 mg mL^−1^ lysozyme (final concentration) at 37°C for 30 min. Subsequently, 50 μL of a proteinase K solution (0.8 mg mL^−1^) was added, followed by the addition of 50 μL of 10% SDS. Samples were incubated at 55°C for 2 h. Final DNA purification was robotically performed using a Chemagen MSM I instrument with the CMG-1037 DNA saliva kit (Perkin Elmer, Waltham, MA).

Sequencing libraries were prepared and sequencing for all the above samples was performed as described previously (18, 45). Briefly, DNA was sheared to an average size of 350 bp using a Covaris M220 focused-ultrasonicator (catalog no. 4482277; Thermo Fisher Scientific, Waltham, MA) with Microtube-50 AFA fiber tubes in accordance with the manufacturer’s instructions. Sequencing libraries were prepared using Illumina’s TruSeq Nano LT library preparation kits (Illumina, San Diego, CA). Libraries were sequenced using a 150-bp paired-end NextSeq500/550 high-output v2 reagent kit (Illumina, San Diego, CA).

### Data generation and processing.

The PA_Shallow metagenomic paired-end read sets were trimmed and quality filtered using illumina-utils (88) with the iu-filter-quality-minoche script based on quality filtering suggestions made by Minoche et al. (89). The PA_Shallow metagenomic reads were individually assembled using metaSPAdes version 3.13.0 with the default parameters (90). The PA_Deep metagenomic paired-end read sets were processed as previously reported (20). The FL metagenomic paired-end read sets were trimmed and quality filtered using the bbduk script from BBMap 38.22 (https://sourceforge.net/projects/bbmap/) in two passes. The first pass used parameters “ktrim=r k = 23 mink = 11 hdist = 1 tbo tpe tbo tpe” for Illumina sequencing adapters, and the second pass used parameters “k = 27 hdist = 1 qtrim=rl trimq = 17 cardinality=t mingc = 0.05 maxgc = 0.95” to remove phiX, low-quality bases, and sequences with unrealistically high or low GC. Additional low-quality bases and sequences were removed with Trimmomatic 0.38 (parameters: LEADING:10 TRAILING:10 MINLEN:100) (91). Unpaired reads were removed using the dropse command from seqtk 1.2 (https://github.com/lh3/seqtk). The cleaned reads from each sample were assembled using SPAdes 3.11.1 (parameters: -meta -k 21,33,55,77,99,127) (90).

### Recovery of the MAGs.

Mapping of quality reads was performed using CoverM v0.4.0 with default parameters (https://github.com/wwood/CoverM). For each assembled metagenome, metagenome-assembled genomes were recovered using MetaBAT1 v0.32.5 (92) with all the sensitivity settings, MetaBAT2 v2.12.1 (93), MaxBin2 v2.2.6 (94) using the 40 and 107 gene sets, and CONCOCT (95) under the default settings. The MAGs were filtered to remove contigs below 1,500 bp. The resulting MAGs from each assembly were assessed for quality and dereplicated using DAStool with default settings (96). Completeness and contamination rates of the MAGs were assessed using CheckM v1.0.13 (97) with the “lineage wf” command. MAGs from a single sample type were subsequently dereplicated using dRep v2.2.3 (98) using the dereplicate_wf at ≥97% average nucleotide identity over ≥70% alignment, and the representative MAGs were chosen based on genome completeness. The dereplicated MAGs were further refined by reassembling the mapped quality trimmed reads with SPAdes (99) using the –careful and –trusted-contigs setting. Additional scaffolding and resolving of ambiguous bases of the MAGs were performed using the “roundup” mode of FinishM v0.0.7 (https://github.com/wwood/finishm).

MAGs were classified into five sample types. FL MAGs were classified as FL_Shallow, FL_Mid, and FL_Deep based on their relative abundances at different depths revealed by read mapping. MAGs were classified as FL_Shallow when they were most abundant at 150 m or shallower, as FL_Mid at 175 m to 1,000 m, and as FL_Deep at 4,000 m. SPA MAGs that were recovered from the 150-m sediment traps (48) were classified as PA_Shallow. Since surface-water microbes were also exported on sinking particles that reached 4,000 m (20), MAGs recovered from the 4,000-m SPA microbes (PA_Deep) that contained surface-associated genes (including those encoding proteorhodopsins, chlorophyll/bacteriochlorophyll *a* synthase, and deoxyribodipyrimidine photolyase) were reclassified as PA_Shallow (see Methods) (Table S2), since these originated from the export of surface-water-derived sinking particles (20).

Sixty-one 4,000-m PA_Deep MAGs were reannotated as PA_Shallow based on their photic zone-dependent gene representation, well documented physiologies and lifestyles (e.g., oxygenic and anoxygenic photoautotrophs and photoheterotrophs), and/or depth profile read mapping densities. These included the following MAGs: DT-Alcanivorax-1, DT-Alteromonas-1, DT-Bdellovibrionales-2, DT-Bdellovibrionales-3, DT-Caenarcaniphilales-1, DT-Chromohalobacter-1, DT-Cognatishimia-1, DT-Coraliomargarita-1, DT-Dinoroseobacter-1, DT-Ekhidna-1, DT-Epibacterium_A-1, DT-Erythrobacter-1, DT-Erythrobacter-2, DT-Flavobacteriaceae-1, DT-Flavobacteriaceae-3, DT-Flavobacteriaceae-4, DT-Flavobacteriales-4, DT-Gilvibacter-1, DT-Halieaceae-1, DT-Halioglobus-1, DT-Halomonas-1, DT-Halomonas-3, DT-Henriciella-1, DT-Henriciella-2, DT-Henriciella-3, DT-Hyphomicrobiaceae-1, DT-Idiomarina-1, DT-Idiomarina-3, DT-Ilumatobacteraceae-1, DT-Legionellales-1, DT-Micavibrionaceae-1, DT-Mycoplasmatales-1, DT-Mycoplasmatales-2, DT-Oleispira-1, DT-Oligoflexales-1, DT-Parvularculaceae-1, DT-Phaeodactylibacter-1, DT-Phycisphaerales-1, DT-Pseudoalteromonas-1, DT-Pseudobacteriovorax-1, DT-Pseudomonadales-2, DT-Psychroserpens-1, DT-Psychrosphaera-1, DT-Rhizobiales-4, DT-Rhodobacteraceae-1, DT-Rhodobacteraceae-2, DT-Rhodobacteraceae-3, DT-Richelia-1, DT-Rickettsiaceae-1, DT-Rickettsiales-1, DT-Rivularia-1, DT-Salinicola-1, DT-Saprospiraceae-1, DT-Saprospiraceae-2, DT-Shewanella-1, DT-Simkaniaceae-1, DT-Verrucomicrobiales-1, DT-Vibrio-1, DT-Winogradskyella-1, DT-Winogradskyella-2, DT-Xanthomonadales-1, and DT-Crocosphaera.

### Taxonomic inference of the MAGs.

Classification of the MAGs was determined using GTDB-Tk (48) v.1.3.0 implementing the classify_wf command (https://github.com/Ecogenomics/GTDBTk). Briefly, marker genes were identified in each genome, aligned, concatenated, and classified with pplacer to identify the maximum-likelihood placement of each genome’s concatenated protein alignment in the GTDB-Tk reference tree. GTDB-Tk classifies each genome based on its placement in the reference tree, its relative evolutionary distance, and FastANI distance.

### Functional annotations.

For all MAGs, genes were called and annotated using Prokka v.1.13 (100). Additional annotation was performed using the blastp “very sensitive” setting in Diamond v0.9.30.131 (101) (https://github.com/bbuchfink/diamond.git) against UniRef100 (accessed September 2019) (102), clusters of orthologous groups (COG) (103), and Pfam 31 (104) and TIGRfam 15.0 hidden Markov models (HMMs) (105). Genes were assigned KEGG Ontology (KO) IDs by hmmsearch against KOfam with predefined thresholds (106).

### Calculation of encoded protein elemental composition.

Amino acid sequences from the dereplicated genome set were used to calculate N-ARSC and C-ARSC values using custom scripts from Mende et al. (44) and available at https://github.com/JessAwBryant/gene-characteristics.

### CAZy annotation.

Genes were annotated against dbCAN (3.0) with default parameters (107) and filtered to retain hits with E values of <1e−102 and HMM coverage of ≥0.35. Filtered genes were further classified to their subfamily level, and their corresponding EC numbers were determined using eCAMI (107). SignalP v6.0 (108) was used to detect the presence of signal peptides in genes encoding putative CAZymes.

### Peptidase annotation.

Genes were annotated using the blastp “very sensitive” setting in Diamond v0.9.30.131 (100) against the MEROPS database v12.2 (109) and filtered to retain hits with E values of <1 × 10^−10^. SignalP v5.0 (108) was used to detect the presence of signal peptides in genes encoding putative peptidases.

### Histidine kinase annotation.

Genes were annotated via hmmsearch using protein family (Pfam) domain annotations (104, 110). Histidine kinases were identified based on hits to pfam02518, pfam13581, pfam13589, pfam14501, and pfam07536. Putative histidine kinases with hits to DNA gyrase (pfam00204), HSP90 (pfam00183), and MutL (pfam13941) were filtered out. Genes encoding response regulator receivers were identified based on hits to pfam0072.

### Extracellular secretion system annotations and analyses.

Genes encoding the bacterial secretion systems were predicted using MacSysFinder (111) and the TXSScan reference database (v1.0rc1) (112).

### Virus annotations and analyses.

Putative prophages within the MAGs were identified using VirSorter2 v2.2.3 (113), and their quality was assessed based on checkV (114) v0.8.1. False-positive screening of prophages was performed based on the Sullivan lab’s protocol (https://www.protocols.io/view/viral-sequence-identification-sop-with-virsorter2-5qpvoyqebg4o/v3). Prophage genes were annotated using DRAM-v.py v1.2.4 (115), and phage-associated marker genes encoding CI repressor, phage capsid proteins, phage excisionase, and phage tail proteins were identified.

### Comparative growth efficiencies estimated via CUB-based maximal growth rate metrics.

Maximal growth rates of MAGs based on codon usage bias metrics were predicted using the R package gRodon (84). Different temperature correction settings were applied to the MAGs as follows: PA_Deep and FL_Deep, 2°C; PA_Shallow and FL_Shallow, 24°C; FL-Mid, 10°C. The Gaussian mixture model shown in Fig. 6 was fitted using the mclust v5.4.9 package in R (116).

### Identification of orthologous proteins.

Homologous proteins across all MAGs were identified with OrthoFinder (117) v2.3.3 using default parameters.

### Principal-coordinate analysis.

The presence/absence of each KO ID or orthologous family in each genome was used as input to a nonmetric multidimensional scaling (NMDS) ordination analysis with the Jaccard distance matrix using the metaMDS function in the Vegan v2.6-2 package in R (118, 119).

### Calculation of relative abundance.

To calculate the relative abundance, reads from each metagenomic data set were mapped to the dereplicated MAGs using CoverM v0.3.1 with the “contig” command, a cutoff of 95% minimum identity, and a minimum aligned read length of 75% of each read. Coverage of each contig was calculated with the CoverM “trimmed_mean” option, and the coverage for each MAG was calculated as the average of all contig coverages, weighted by their length. The relative abundance of each MAG in each metagenomic data set was calculated as its coverage divided by total reads in the sample multiplied by 100,000,000.

### Gene enrichment analysis.

The presence/absence of each KO ID or orthologous family in each genome was used as input for the program Scoary (52) to identify significant correlations between gene presence/absence and sample types.

### Statistical analysis.

The mean statistical significance of different proteins of interests and genomic characteristic metrics between sample types were determined through one-way analysis of variance (ANOVA), followed by the Tukey test using the R package multicompview v0.1-8 with the Tukey honestly significant different (HSD) function. All *P* values that were affected by multiple testing were corrected for false discovery using the Benjamini-Hochberg procedure.

### Calculation of amino acid identity.

Average amino acid identity (AAI) between the genomes was calculated using orthologous genes identified through reciprocal best BLAST hits by use of compareM v0.0.5 (https://github.com/dparks1134/CompareM).

### Data visualization.

Figures were generated using pheatmap (120) and ggplot2 (121) packages in R (122). Trees were visualized in ARB (123) and presented using iTOL (124). Venn diagrams were generated using the Venn diagram tool (http://bioinformatics.psb.ugent.be/webtools/Venn/). Figures were further refined using Adobe Illustrator.

### Data availability.

All data were deposited in the NCBI SRA archive as follows. Deep 4,000-m-trap metagenomes and MAGs are deposited under NCBI BioProject no. PRJNA482655, and assembled MAGs are available under NCBI BioSample no. SAMN14675689 to SAMN14675809. Metagenomic reads produced from DNA extracted from shallow 150-m sediment trap samples (collected in 2015) (48) and PA_Shallow MAGs are deposited under NCBI BioProject no. PRJNA358725. For Station ALOHA 0.2-μm-pore-size filter-collected metagenomes/MAGs, read sequence data and FL MAGs are available at NCBI SRA under BioProject no. PRJNA352737, and assemblies can be found under BioSample no. SAMN12604809.
