# Supplementary material for: Diverse Genomic Traits Differentiate Sinking-Particle-Associated versus Free-Living Microbes throughout the Oligotrophic Open Ocean Water Column
Source: mBio. 2022 Jul 12;13(4):e01569-22. doi: 10.1128/mbio.01569-22 (PMC9426571; doi:10.1128/mbio.01569-22)
Supplement: FIG S1 [file mbio.01569-22-sf001.pdf]

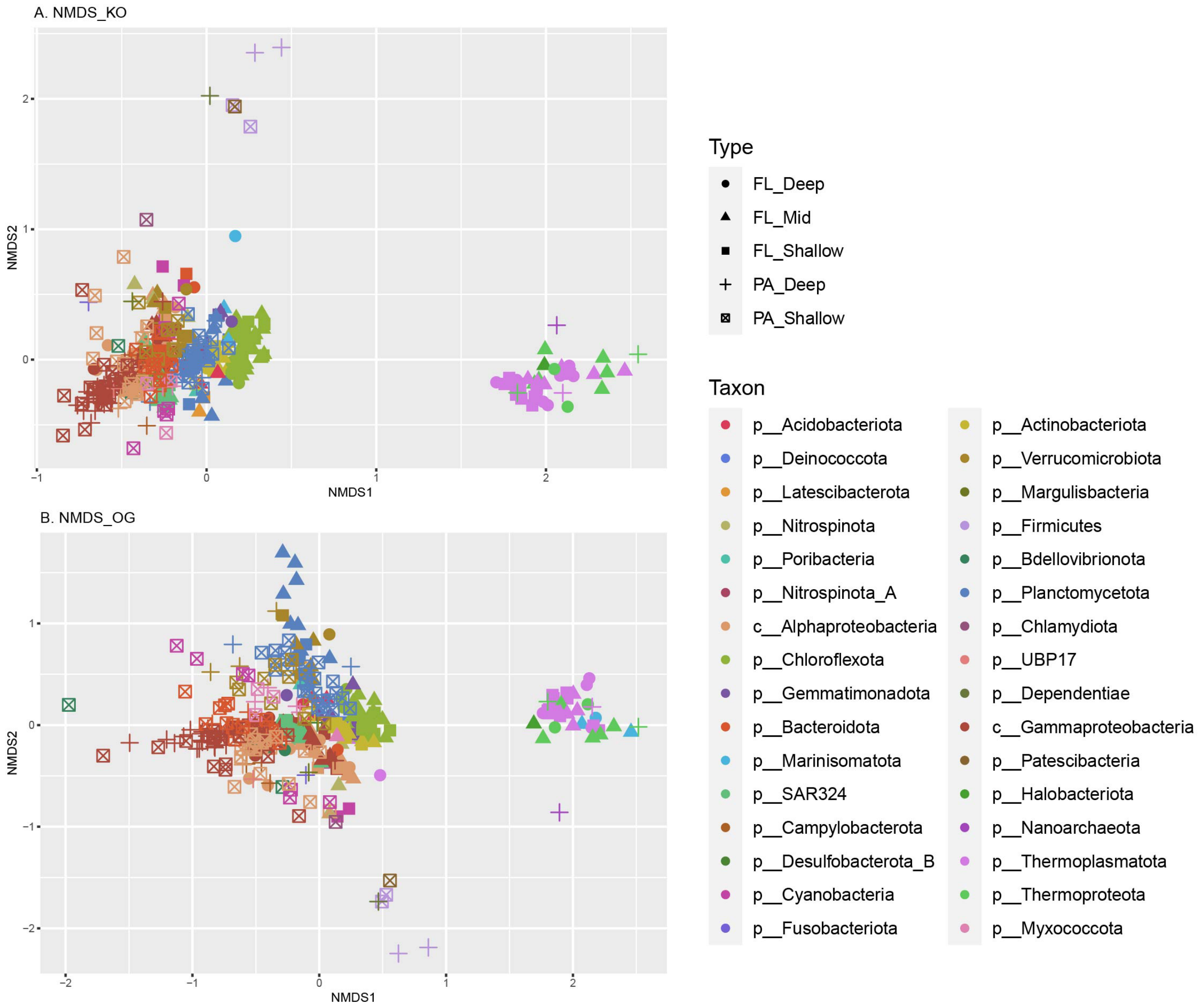

Supplementary Figure 1. Nonmetric multidimensional scaling (NMDS) plots of the recovered MAGs based on gene annotations. A. The NMDS plot was generated based on presence/absence of KEGG Orthology (KO) annotations. B. NMDS plot generated based on presence/absence of orthologous protein families. Shapes indicated the sample type designation of the MAG. Color indicates the taxonomic classification of the MAG. PA and FL designations are as indicated in Figure 1.
