# Supplementary material for: Diverse Genomic Traits Differentiate Sinking-Particle-Associated versus Free-Living Microbes throughout the Oligotrophic Open Ocean Water Column
Source: mBio. 2022 Jul 12;13(4):e01569-22. doi: 10.1128/mbio.01569-22 (PMC9426571; doi:10.1128/mbio.01569-22)
Supplement: FIG S2 [file mbio.01569-22-sf002.pdf]

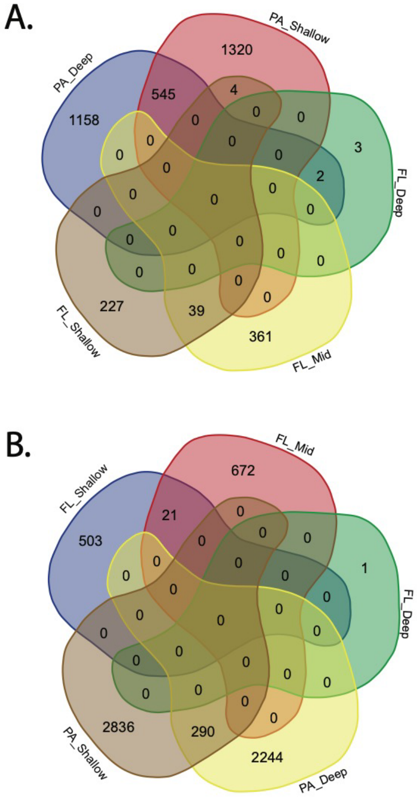

Suppl. Figure 2. Venn diagram of significantly enriched genes. A, B. Venn diagrams of significantly enriched genes based on KEGG Orthology (KO) annotations and orthologous protein family. Significantly enriched KOs and orthologous gene families were determined using Scoary with a q-values less than 0.05 for Fisher's exact test.
