# Supplementary material for: Diverse Genomic Traits Differentiate Sinking-Particle-Associated versus Free-Living Microbes throughout the Oligotrophic Open Ocean Water Column
Source: mBio. 2022 Jul 12;13(4):e01569-22. doi: 10.1128/mbio.01569-22 (PMC9426571; doi:10.1128/mbio.01569-22)
Supplement: FIG S3 [file mbio.01569-22-sf003.pdf]

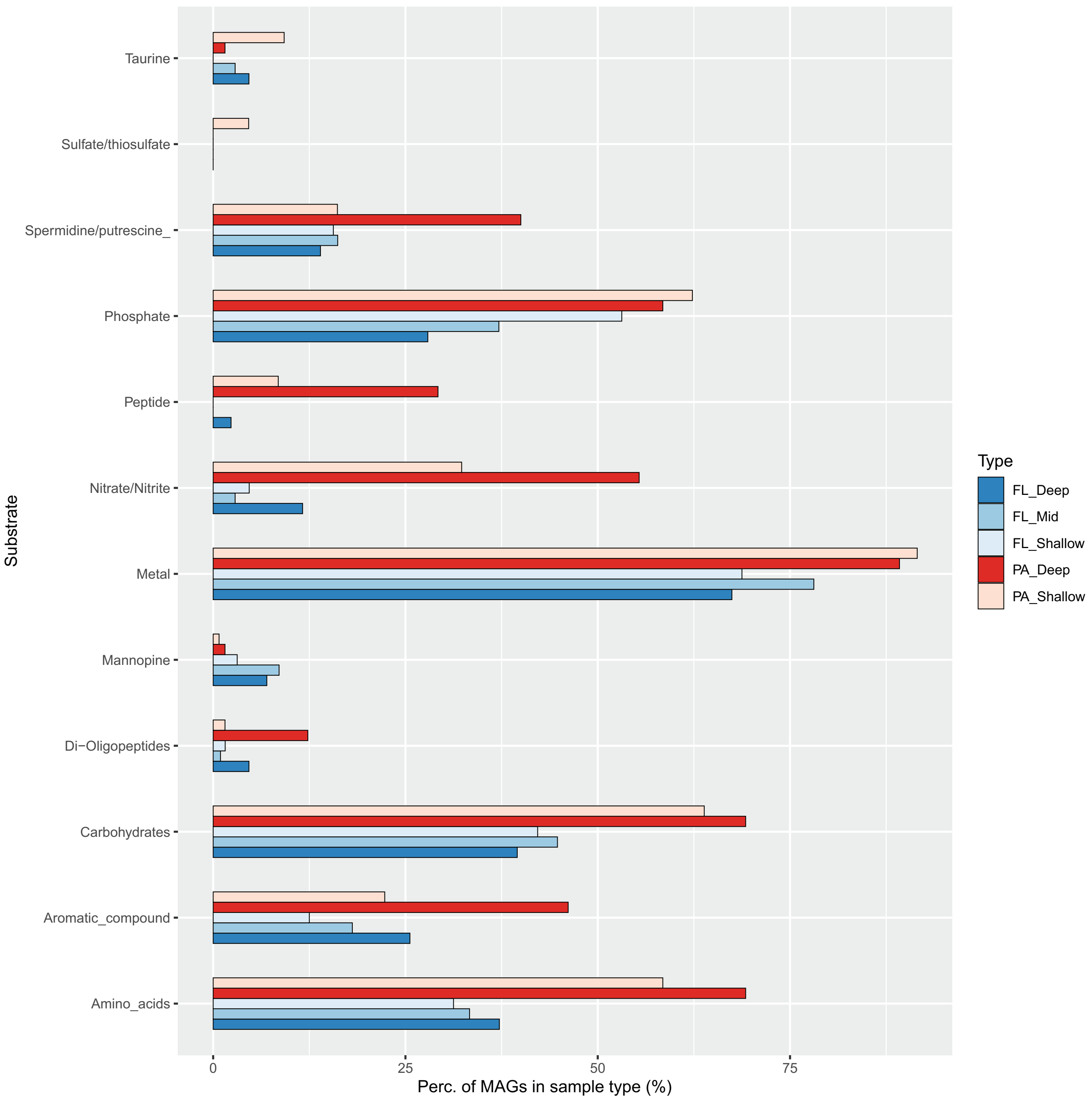

Supplementary Figure 3. Counts of MAGs encoding substrate specific transporters. Genes encoding putative transporters were grouped based on their substrate specificity.
