# Supplementary material for: Diverse Genomic Traits Differentiate Sinking-Particle-Associated versus Free-Living Microbes throughout the Oligotrophic Open Ocean Water Column
Source: mBio. 2022 Jul 12;13(4):e01569-22. doi: 10.1128/mbio.01569-22 (PMC9426571; doi:10.1128/mbio.01569-22)
Supplement: FIG S4 [file mbio.01569-22-sf004.pdf]

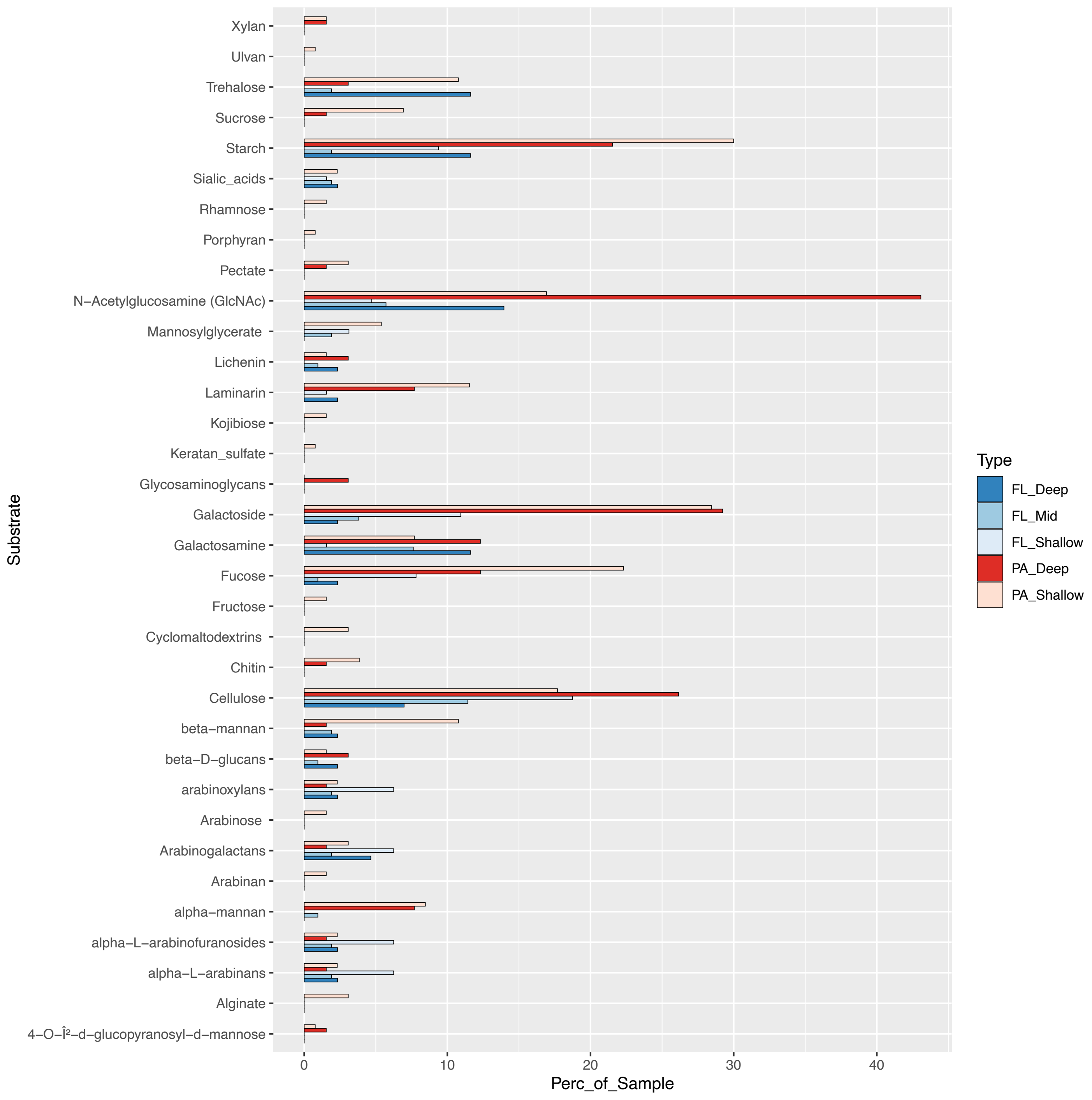

Supplementary Figure 4. Counts of MAGs encoding substrate specific CAZymes. Genes encoding putative CAZymes were annotated against dbCAN (3.0) with default parameters (107), and filtered to retain hits with e values <1e-102, and HMM coverage of ≤ 0.35. Filtered genes were further classified to their subfamily level and their E.C. numbers determined using eCAMI.
