# Supplementary material for: Diverse Genomic Traits Differentiate Sinking-Particle-Associated versus Free-Living Microbes throughout the Oligotrophic Open Ocean Water Column
Source: mBio. 2022 Jul 12;13(4):e01569-22. doi: 10.1128/mbio.01569-22 (PMC9426571; doi:10.1128/mbio.01569-22)
Supplement: FIG S5 [file mbio.01569-22-sf005.pdf]

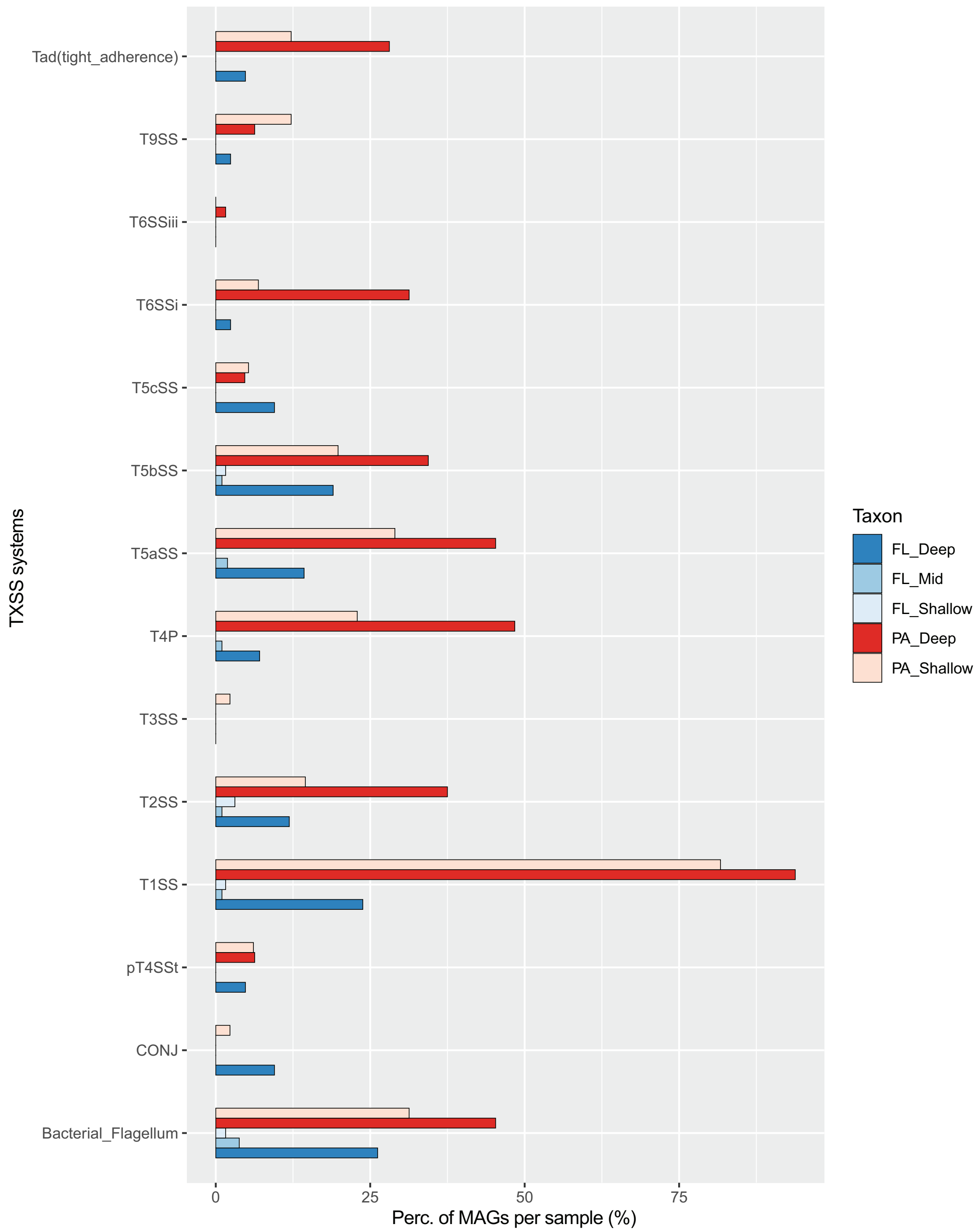

Supplementary Figure 5. Frequency of different extracellular secretion systems in particle associated versus free-living bacterial genomes in the water column. Genes encoding bacterial secretion systems were predicted using MacSysFinder with the TSScan reference database (v1.0rc1).
